# Reliability and validity of the Japanese version of the Self-Stigma Scale in patients with type 2 diabetes

**DOI:** 10.1186/s12955-014-0179-z

**Published:** 2014-12-12

**Authors:** Asuka Kato, Misato Takada, Hideki Hashimoto

**Affiliations:** Division of Social Medicine, Graduate School of Medicine, The University of Tokyo, 7-3-1 Hongo, Bunkyo-ku, Tokyo 113-0033 Japan; Department of Health and Social Behavior, School of Public Health, The University of Tokyo, 7-3-1 Hongo, Bunkyo-ku, Tokyo 113-0033 Japan

**Keywords:** Self-stigma, Psychometric assessment, Reliability, Validity, Type 2 diabetes, Japanese

## Abstract

**Background:**

Self-stigma has been highlighted and researched in relation to patients with chronic illnesses, as it may have a negative impact on their treatment adherence. However, self-stigma has not yet been investigated in patients with type 2 diabetes. In order to evaluate the extent to which patients with type 2 diabetes experience self-stigma, which may result in their poor self-care management, there is a need for a specific tool to measure self-stigma in patients with type 2 diabetes. This study assessed the psychometric properties of a Japanese version of the Self-Stigma Scale (SSS-J) in patients with type 2 diabetes.

**Methods:**

The reliability and validity of the SSS-J were evaluated using a consecutive sample of 210 outpatients with type 2 diabetes from university hospitals and from hospitals or clinics specializing in diabetes treatment. Confirmatory factor analysis was conducted to assess the factors theorized by the original Self-Stigma Scale. Cronbach’s alpha for internal reliability and Pearson’s correlations for construct validity were used for evaluation of psychometric properties. Pearson’s correlations for test-retest reliability of the SSS-J were also performed.

**Results:**

Confirmatory factor analysis verified the three-factor structure of the SSS-J, consisting of cognitive, affective, and behavioral subscales. The model fit indices were as follows: the goodness-of-fit index was 0.78, the adjusted goodness-of-fit index was 0.70, the comparative fit index was 0.88, and the root mean square error of approximation was 0.07. Cronbach’s alpha of the SSS-J was 0.96 (cognitive: alpha = 0.92; affective: alpha = 0.93; behavioral: alpha = 0.83). The SSS-J was associated with self-esteem (r = −0.43, p < 0.01), self-efficacy (r = −0.38, p < 0.01), and depressive symptoms (r = 0.39, p < 0.01). The 2-week test-retest reliability demonstrated satisfactory stability (r = 0.76, p < 0.01).

**Conclusions:**

The SSS-J is reliable and valid for assessment of the extent of self-stigma in Japanese patients with type 2 diabetes.

## Introduction

Self-stigma is experienced by individuals who have negative attitudes towards themselves as a result of their condition and/or characteristics [1,2]. Self-stigma is also referred to as internalized stigma. Conversely, public stigma represents negative reactions of the general public towards a group based on stereotypical attributes that distinguish that group in society [1,2]. Public stigma is also known as social stigma. Self-stigma has a negative impact on individuals, resulting in decreased self-esteem, self-efficacy, life satisfaction, social adaptation, overall well-being, and social networking [1,3-7]. Additionally, this may lead to either treatment avoidance or diminished treatment adherence in patients with chronic illnesses [4,8,9].

Chronic illness, in particular type 2 diabetes, requires a considerable amount of self-management by patients in their everyday lives. Additionally, type 2 diabetes is one of the most common chronic illnesses, and its prevalence has dramatically increased worldwide in the past two decades [10]. Likewise, the prevalence of type 2 diabetes in Japan has been on the rise [11]. As the number of patients with type 2 diabetes increases, some preconceived ideas about their particular characteristics result in blame being placed on them, because their condition is considered to be a lifestyle-related disease. People with type 2 diabetes are often subject to stigmatizing attitudes from the general population. Recently, public stigma has been highlighted and researched in relation to type 2 diabetes. According to these studies, public stigma has a negative impact on diabetes self-care management [12-15]. However, previous studies have shown that merely perceiving public stigma does not necessarily lead to self-stigma [1,2]. Self-stigma is the issue that will impact patients’ behavioral goals through decreased self-esteem, self-efficacy, and psychological well-being [1,4,7,9]. As a result, patients become reluctant to seek necessary treatment and there is a reduced treatment adherence [4,8,9]. Therefore, it is extremely important to assess the extent to which patients experience self-stigma, so that early medical interventions for self-esteem and self-efficacy can be provided to avoid suboptimal treatment outcomes. Nevertheless, there has not yet been a study on self-stigma in patients with type 2 diabetes and how self-stigma could potentially have an impact on their treatment outcomes.

To assess the extent to which patients with type 2 diabetes experience self-stigma, there is a need for a validated tool to measure this construct. Although a number of validated tools for self-stigma have been developed, most have only focused on mental disorders [16-19]. The Self-Stigma Scale was originally developed to quantify and evaluate concealed self-stigma among various groups of minorities, such as immigrants and sexual minorities, as well as mental health patients [20]. Type 2 diabetes is one of the conditions that cannot be detected by looking at patients’ physical appearance. Therefore, patients with type 2 diabetes can hide their stigmatized condition from the mainstream of healthy individuals, although they do so with fear of being discovered [14,15]. For this reason, the Self-Stigma Scale is viewed as the best tool that can be easily adapted for use with the particularly concealable condition of type 2 diabetes. In this study, we translated the Self-Stigma Scale from English to Japanese and examined its reliability and validity in patients with type 2 diabetes. Furthermore, we tested the equivalency between the Self-Stigma Scale (hereafter defined as “the original scale”) and the Japanese version (SSS-J) when assessing patients with type 2 diabetes.

## Methods

### Development of the SSS-J

**Figure 1:**
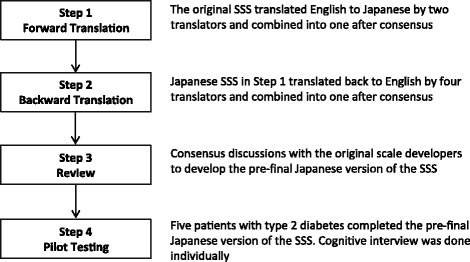
**Flow chart describing the development of the Japanese version of the Self-Stigma Scale.** SSS: Self-Stigma Scale.

Translation procedures were based on the Consensus-based Standards for the Selection of Health Measurement Instruments checklist [21]. Four steps were followed (Figure 1). Multiple forward and backward translations were performed by six translators. They were selected according to the following criteria:

1. There were two forward translators who were both native Japanese speakers. One translator had expertise on stigma, and the other was a language expert but lacked knowledge about stigma.
2. There were four backward translators. Two were native English speakers, and the other two were native Japanese speakers. They were all language experts with no knowledge of stigma or the original scale.

In Step 1, two native Japanese speakers worked independently and translated the original scale into Japanese. They were fully informed about the objectives of the whole translation procedure, and forward translated all the questionnaire items, not word-for-word but with emphasis on the meaning of each item. We then combined these two Japanese translations into one. To amalgamate these Japanese translations, the two translators and three authors had discussions to reach a consensus in terms of the following four criteria: content, semantics, conceptualization, and technical equivalence with the original scale, as well as cultural adaptations. In Step 2, another two native Japanese speakers worked independently and were asked to backward translate the Japanese translation in Step 1 into English. We then combined these two English translations into one by reaching a consensus with the two translators in the same way as in Step 1. Afterwards, two bilingual individuals, whose native language was English, checked for any semantic discrepancies between the Japanese translation in Step 1 and the backward translation in Step 2. All these backward translators were blinded to the original scale. In Step 3, the English translation produced in Step 2 was reviewed by the original scale developers. Based on suggestions from them, some questionnaire items were revised through repeated forward and backward translation procedures (Steps 1 and 2) to reflect the original meaning after translation.

In Step 4, the translation was pretested in cognitive interviews by five outpatients with type 2 diabetes to ensure accessibility and comprehension. They were men and women aged 30–74 years with different educational backgrounds ranging from those without a high school education to those with a bachelor’s degree. According to their comprehension level of the Japanese translation, some words were altered to even plainer language. Additionally, Japanese patients with type 2 diabetes did not understand the direct translation of the term “identity”. Therefore, with permission of the original scale developers, we replaced the term with a Japanese phrase, “oneself with the illness, diabetes”, while maintaining the conceptual equivalence in the original scale. Thereafter, we processed Steps 2 and 3 in the same way. Finally, we obtained permission from the original scale developers to field test the revised translation.

### Testing the SSS-J in type 2 diabetes patients

The SSS-J was tested between November 2013 and March 2014. Consecutive sampling was used to recruit all outpatients with type 2 diabetes who visited a diabetologist on a specific date at four locations, comprising two university hospitals (The University of Tokyo Hospital and Teikyo University Hospital), one non-university affiliated hospital (Mitsui Memorial Hospital), and one non-university affiliated clinic (The Institute for Adult Diseases Asahi Life Foundation) in Japan. The following patients were excluded: non-native Japanese speakers; those aged 75 years or older; and those with a serious mental disorder, such as dementia, that affected their cognition. Additionally, patients who required urgent medical procedures or examinations were excluded.

During enrollment, the purpose of the study was explained by study staff, and informed consent was obtained from those who agreed to the terms of the study. The SSS-J was self-administered in the majority of the participants. For those who had either visual loss or poor literacy skills, an audiotape was provided to read out each question. To determine the test-retest reliability, all participants took home another SSS-J questionnaire to complete after 2 weeks and this was mailed back to our office. Reminder phone calls were made up to two times as necessary.

The sample size was calculated based on the number required to perform the factor analysis for the psychometric assessment of the scale. Because it had 39 items, the minimal sample size was 195 based on a participant-to-item ratio of 5:1 [22].

This study was approved in advance by the Research Ethics Committee of The University of Tokyo Graduate School of Medicine and Faculty of Medicine.

### Measures

A self-administered questionnaire was used to assess diabetes-related complications and hemoglobin A1c. The number of complications was calculated with reference to the Diabetes Complications Index (DCI) [23]. The score ranged from 0 to 6. Participants were asked to fill out their hemoglobin A1c levels based on a copy of laboratory results received that day.

### SSS-J scale

The SSS-J comprises 39 items that allow four responses in a Likert scale: strongly disagree, disagree, agree, and strongly agree. The responses are afforded a score of 0, 1, 2, and 3, respectively. The total possible scores have a range of 0–117. A higher score represents a higher level of self-stigma.

We predicted that the SSS-J would be negatively associated with several self-identity measures, such as self-esteem and self-efficacy, and that it would also be related to greater levels of depressive symptoms. Our predictions in a Japanese sample of patients with type 2 diabetes were informed by the results of a previous study [20]. For comparison, participants completed the following measures in addition to the SSS-J.

### Self-esteem

The Rosenberg Self-Esteem Scale was used to assess the level of self-esteem [24,25]. It is a widely accepted scale because of its high reliability and validity. It contains 10 items scored on a 4-point Likert scale from 1 (strongly disagree) to 4 (strongly agree). Five negative items were reverse-scored to compute the total scores of individual participants. In this study, it had an internal consistency of 0.79.

### Self-efficacy

The General Self-Efficacy Scale was applied to assess individual strength in general self-efficacy across a variety of settings in everyday life [26]. It is reliable and valid, and is commonly used to measure self-efficacy in Japan. It is a 16-item scale using dichotomous (yes/no) questions. In this study, it had an internal consistency of 0.84.

### Depressive symptoms

The nine-item depression module of the Patient Health Questionnaire (PHQ-9) was used to assess depressive symptoms during the previous 2 weeks [27,28]. It is a reliable and valid measure of depression severity for clinical use. Each item is scored on DSM-IV (*Diagnostic and Statistical Manual of Mental Disorders*-IV) criteria from 0 (not at all) to 3 (nearly every day). In this study, it had an internal consistency of 0.86.

### Statistical analysis

The mean and standard deviation of each item of the SSS-J were determined. Cronbach’s coefficient alpha was calculated to assess the internal reliability of each subscale defined by the original scale. Confirmatory factor analysis was conducted on the SSS-J to confirm that the three-factor model theorized in the original scale would achieve the best fit for the data in Japanese patients with type 2 diabetes. Model fitness was assessed based on the maximum likelihood method by using the following fit indices: goodness-of-fit index (GFI), adjusted goodness-of-fit index (AGFI), comparative fit index (CFI), and root mean square error of approximation (RMSEA). The model was built using three self-stigma subscales, which consisted of 19 cognitive, 14 affective, and six behavioral items as observed variables. The construct validity was examined with Pearson’s correlations in the Self-Esteem, General Self-Efficacy, and PHQ-9 scales.

All analyses were conducted with SPSS version 18.0 (SPSS Japan Inc., Tokyo, Japan), except for the confirmatory factor analysis, which was performed using AMOS version 18.0 (SPSS Japan Inc., Tokyo, Japan).

## Results

Physicians recruited 259 patients with type 2 diabetes and written informed consent was obtained from 218, giving a response rate of 84.2%. Of these patients, 217 completed the questionnaire (one patient declined). The percentage of missing data was zero for all questionnaire items. In the analysis, we excluded five participants who answered all 39 items of the SSS-J with a “strongly disagree” response, because they responded strongly to stigma, and we did not know whether the scale could measure what it was originally intended to assess. We also excluded two participants who had vision loss and completed the questionnaire with the aid of an audiotape, because they used different cognitive tasks from those who were able to answer all of the questionnaire items on their own. Therefore, 210 participants were included in our final analysis. Of these remaining participants, 187 answered and returned the second questionnaire containing the SSS-J items 2 weeks later, producing a response rate of 89.0%.

### Descriptive statistics for the SSS-J

The sociodemographic and clinical characteristics of the participants are shown in Table 1. There were 169 male participants (80.5%) and 41 female participants (19.5%), and the mean age was 60.1 ± 10.0 years. The mean duration of type 2 diabetes was 13.3 ± 9.6 years and the mean hemoglobin A1c level was 7.3 ± 1.2%. The number of complications was calculated as the simple sum of the six complications from the DCI [23]. The score range was 0–6, and 62.4% of participants had no complications. Table 2 indicates the means and standard deviations for each item of the SSS-J questionnaire. In the SSS-J, the mean scores were lower and the standard deviations were smaller in our patients for all items compared with the scores for mental health patients using the original scale. The score distributions were not found to be highly skewed.

**Table 1:** **Sociodemographic and clinical characteristics of participants (n = 210)**

**Patient characteristics**	**N (%) or mean (** **±SD)**
**Sex:**	
Men	169 (80.5)
Women	41 (19.5)
**Age (years):**	60.1 (± 10.0)
**Marital status:**	
Married	152 (72.4)
Unmarried/Divorced/Bereaved	58 (27.6)
**Employment:**	
Full-time work	121 (57.7)
Part-time work	50 (23.8)
Retired/not working	36 (17.1)
Others	3 (1.4)
**Highest education:**	
Have not graduated high school	16 (7.6)
High school	66 (31.4)
Technical/junior college	34 (16.2)
Bachelor’s degree or higher	94 (44.8)
**Duration of diabetes (years):**	13.3 (± 9.6)
**Primary treatment:**	
Oral hypoglycemic agents	123 (58.6)
Insulin injections	15 (7.1)
Insulin injections and oral hypoglycemic agents	45 (21.4)
Other injectable medications (other than insulin)	14 (6.7)
Lifestyle	13 (6.2)
**HbA1c (%):**	7.3 (± 1.2)
**Numbers of diabetes-related complications:**	
0	131 (62.4)
1	53 (25.2)
2	16 (7.6)
3	9 (4.3)
4	1 (0.5)

SD: standard deviation; HbA1c: glycated hemoglobin.

**Table 2:** **Scores of items in the Japanese version of the Self-Stigma Scale in type 2 diabetes patients**

		**M**	**SD**
**COGNITIVE**	Being a ___ takes away many opportunities from me. (29)	1.85	0.67
	I think that I am less competent than ordinary people because I am a ___. (26)	1.59	0.57
	I feel that my life is unenjoyable because of myself with the illness, ___. (31)	1.81	0.69
	No matter how hard I work, I cannot match others because of myself with the illness, ___. (30)	1.64	0.55
	Who I am: having the illness, ___, is a heavy burden to me. (38)	2.17	0.78
	I have low expectations in life because I am a ___. (18)	1.80	0.64
	I am not qualified to compete with others because I am a ___. (27)	1.54	0.54
	Who I am: having the illness, ___, is a stigma in my life. (13)	1.92	0.73
	Who I am: having the illness, ___, has a negative impact on my financial situation. (35)	2.15	0.83
	I am inferior to others because I am a ___. (21)	1.66	0.58
	Who I am: having the illness, ___, causes inconvenience on my daily life. (34)	2.13	0.76
	I cannot measure up to ordinary people because I am a ___. (10)	1.68	0.65
	I cannot change myself with the illness, ___. (11)	1.90	0.67
	I lower my standards of living because I am a ___. (6)	1.90	0.65
	My life is meaningless because I am a ___. (4)	1.63	0.51
	I need assistance from others because I am a ___. (12)	1.77	0.67
	My social interactions are limited because I am a ___. (2)	2.04	0.66
	It is quite normal for me to be alienated by others because I am a ___. (5)	1.55	0.51
	I feel much stressed because I am a ___. (1)	2.49	0.73
**AFFECTIVE**	I cannot feel confident about who I am because of myself with the illness, ___. (33)	1.74	0.61
	I am worried about who I am: having the illness, ___, would create obstacles to me. (25)	1.90	0.74
	I have negative feelings about myself with the illness, ___. (23)	1.79	0.68
	I am unhappy because I am a ___. (32)	1.80	0.69
	I feel helpless because I am a ___. (37)	1.59	0.57
	I am discouraged because I am a ___. (28)	1.84	0.71
	I hate myself because I am a ___. (24)	1.89	0.71
	I get embarrassed because of myself with the illness, ___. (39)	1.68	0.68
	I feel angry because I am a ___. (22)	1.96	0.75
	I feel uncomfortable being a ___. (17)	2.22	0.80
	I feel sorry that I am a ___. (8)	1.98	0.73
	I feel there is nothing I can do about being a ___. (16)	1.92	0.74
	I fear that people around me would find out that I am a ___. (15)	1.75	0.78
	I am ashamed of being a ___. (3)	1.99	0.72
**BEHAVIORAL**	I avoid interacting with others because I am a ___. (36)	1.55	0.56
	I keep my distance from others because I am a ___. (19)	1.57	0.62
	I give up on myself because I am a ___. (14)	1.69	0.63
	I hide myself with the illness, ___. (20)	1.66	0.77
	I make friends only with people who are in the same condition as mine. (9)	1.48	0.56
	I dare not to make new friends because they might find out that I am a ___. (7)	1.53	0.58

____ in each question item was replaced by either “diabetes” or “patient with diabetes”. The numerical values in parentheses after each question item reflect the order in which the items appeared as the patients completed the SSS-J. Questionnaire items within each subscale are listed in descending order of factor loading. M: mean; SD: standard deviation; SSS-J: Self-Stigma Scale, Japanese version.

### Confirmatory factor analysis

**Figure 2:**
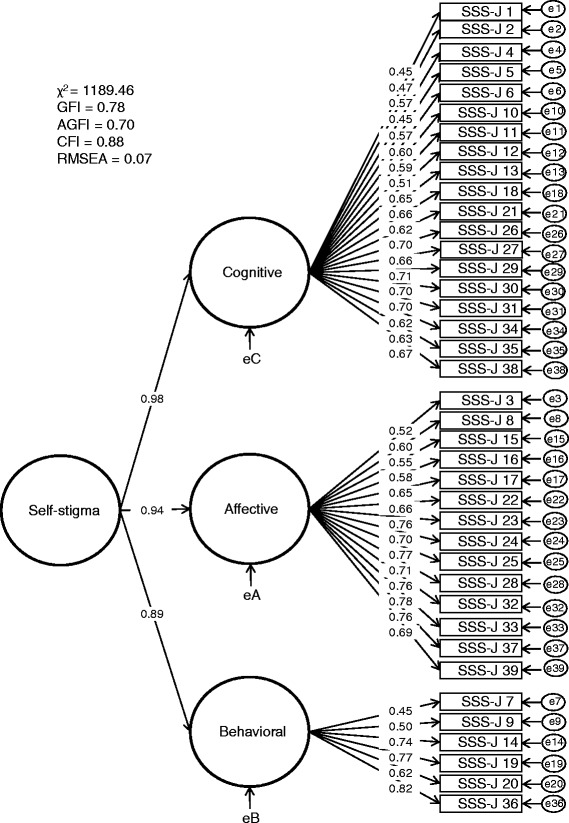
**Results of the confirmatory factor analysis of the Japanese version of the Self-Stigma Scale in patients with type 2 diabetes.** GFI: goodness-of-fit index; AGFI: adjusted GFI; CFI: comparative fit index; RMSEA: root mean square error of approximation.

The results of the confirmatory factor analysis are demonstrated in Figure 2. All path coefficients were significant. The model fit indices were as follows: GFI = 0.78, AGFI = 0.70, CFI = 0.88, and RMSEA = 0.07. The goodness-of-fit indices for the confirmatory factor analysis were acceptable. There was a relatively good fit between the three-factor model and the observed data. The GFI in this sample was below 0.9. However, the GFI depends on the total number of observed variables [29]. As the SSS-J was tested using 39 items, the GFI in this sample would be less than 0.9. However, all factor loadings based on the three-factor model of the 39 items were higher than the general standard (0.4) in this sample. Additionally, the CFI value of 0.88 is close to 0.90, indicating a relatively good fit [30]. The RMSEA value of 0.07 is in the reasonable fit range of 0.05–0.08 [31].

### Internal consistency

The internal consistency of the SSS-J was excellent: Cronbach’s alpha = 0.96. The internal consistency of each conceptual dimension of the SSS-J ranged from acceptable to excellent: cognitive (Cronbach’s alpha = 0.92), affective (Cronbach’s alpha = 0.93), and behavioral (Cronbach’s alpha = 0.83). This demonstrated excellent internal consistency reliability, indicating adequate interrelations between the items of the scale.

### Test-retest reliability

Test-retest reliability was determined by comparing responses to the SSS-J among 187 participants (89.0%) who completed the questionnaire at home after a 2-week interval. The correlation coefficient for test-retest reliability was 0.76 (p < 0.01). This demonstrated acceptable reproducibility. A correlation coefficient range of 0.7–0.8 is acceptable [32].

### Construct validity

Significant Pearson’s correlations were observed when analyzing the scores in the SSS-J and the other comparable scales. The Rosenberg Self-Esteem (r = −0.43, p < 0.01) and the General Self-Efficacy (r = −0.38, p < 0.01) scales were negatively correlated with the SSS-J, whereas the PHQ-9 demonstrated a positive correlation (r = 0.39, p < 0.01). This was consistent with predictions based on the results of the original scale.

For the participants in this study, the median self-stigma score was 75 with a range of 40–109, and the median depressive symptoms score was 3 with a range of 0–25.

## Discussion

This is the first study to examine the reliability and validity of the Japanese version of the Self-Stigma Scale to assess the extent of self-stigma among individuals with type 2 diabetes. The SSS-J was developed based on several forward and backward translations with cross-cultural validation. All the questionnaire items were comprehensible by both elderly people and individuals with lower educational levels. The SSS-J also offered reliable and valid determination of psychometric properties as well as the relevant structure of self-stigma consisting of cognitive, affective, and behavioral subscales in the same manner as the original scale [20].

The results indicated that the SSS-J consistently constructed the three-factor model as in the original scale while using a different focus group and language. Furthermore, they indicated that each subscale had adequate internal consistency. This demonstrates that the SSS-J can be used to assess self-stigma among Japanese people with type 2 diabetes. It should be noted that among people with type 2 diabetes, items with higher factor loadings differed from those in the original scale (Table 2).

In the SSS-J, the mean scores in all questionnaire items were lower among patients with type 2 diabetes than in the original scale that targeted minorities with concealed self-stigma, such as mental health patients. As previous studies have shown, it is speculated that those patients who have a high educational attainment, whose conditions are less severe, and whose treatment is sustained are less likely to develop self-stigma [33-35]. Our sample consisted of a higher percentage of these subgroups. However, we cannot conclude anything definite about the possible reasons for the low scores to explain the current data. Further research is needed to determine whether patients with type 2 diabetes do indeed have lower scores in terms of self-stigma when compared with patients with other chronic physical illnesses, as well as mental illnesses.

There are some limitations to this study. First, the participating patients with type 2 diabetes were recruited from specialist hospitals/clinics. Patients who were seen regularly by a primary care doctor were not included, and neither were those who were not treated after diagnosis. Further research in a more representative population is needed in the specialty field, and also in primary care settings. Second, a sample with similar disease characteristics as used in the original scale, i.e., patients with mental disorders, was not tested. Further research using such a sample is needed to assess the cross-cultural validity of the SSS-J more precisely. Third, diabetes-related complications were self-reported by participants, and the data were likely to be under-reported. To overcome the limitation of this self-reported assessment, an alternative variable, the duration of type 2 diabetes, was used to proxy for the severity of the illness. Fourth, there was a sex imbalance in our sample, as the percentage of men was 80.5% and the percentage of women was 19.5%. Although the prevalence of type 2 diabetes is higher among men (15.3%) than women (7.3%) in Japan [11], it is not known exactly why we had such a large number of male respondents, compared with female respondents. Finally, we were not able to assess the concurrent validity of self-stigma, as no similar validated scale exists for comparison. Although there are other generic stigma scales targeting people with psychiatric disorders, they do not necessarily assess the same construct as self-stigma for people with chronic physical illnesses.

Nevertheless, use of this self-administered scale would be valuable to assess the levels of self-stigma in individual patients with type 2 diabetes in daily clinical practice. In a chronic disease such as type 2 diabetes, patient self-care is necessary and a validated tool of self-stigma would indicate the need for measures to be taken to eliminate a possible barrier to optimal treatment. In psychiatric patients, there is evidence that self-stigma reduction programs are effective in improving their self-esteem, promoting their readiness to change their own problematic behavior, and facilitating their treatment adherence [36-38]. Similar effects may be expected in patients with type 2 diabetes, with improved treatment adherence by lowering levels of self-stigma through patient education programs.

## Conclusions

This study has demonstrated that the SSS-J produces the same factor structure as the original Self-Stigma Scale through testing by confirmatory factor analysis. Furthermore, it indicated that the SSS-J could be a useful clinical tool to help healthcare professionals identify high-risk self-stigma patients with type 2 diabetes. For clinical use of the SSS-J, given its length, a shorter version should be developed as this will avoid some redundant items while incorporating more relevant items. In addition, further studies are needed to discover the optimum time during the treatment process to respond to self-stigma in individual patients with type 2 diabetes, as well as the most effective places to assess the extent of self-stigma. With continual use of the SSS-J, the degree of self-stigma may be accurately assessed. As a result of these assessments, it would be possible to provide patients with different treatment strategies in addition to early intervention to help reduce self-stigma. This could then lead to optimal treatment outcomes.
